# A matter of proportion? Associational effects in larval anuran communities under fish predation

**DOI:** 10.1007/s00442-018-4141-3

**Published:** 2018-04-30

**Authors:** Jan M. Kaczmarek, Mikołaj Kaczmarski, Jan Mazurkiewicz, Janusz Kloskowski

**Affiliations:** 1 Department of Zoology, Institute of Zoology, Poznań University of Life Sciences, ul. Wojska Polskiego 71C, 60-625 Poznań, Poland; 2 Department of Inland Fisheries and Aquaculture, Institute of Zoology, Poznań University of Life Sciences, ul. Wojska Polskiego 71C, 60-625 Poznań, Poland

**Keywords:** Associational resistance, Indirect effects, Mimicry, *Bufo bufo*, *Rana temporaria*

## Abstract

In Batesian mimicry, a species lacking defences against predators benefits from mimicking the aposematic signal of a defended species, while the model may incur the costs of reduced defensive efficacy. Similar reciprocal indirect effects may emerge even when the signal is not mimicked; termed associational effects, such interactions are well known in plants sharing herbivores but have received little attention in animal studies. We investigated associational interactions in a system where unequally defended prey (chemically defended *Bufo bufo* and undefended *Rana temporaria* tadpoles), sharing general morphology but not an aposematic signal, were exposed to predation by the carp *Cyprinus carpio* along a gradient of relative prey abundance. In the absence of fish, the assemblage composition had no effect on the survival of *Rana*, while that of *Bufo* decreased with increasing abundance of *Rana*. Fish reduced the survival of tadpoles from both species. However, increased relative abundance of *Bufo* in the community led to enhanced survival in both *Bufo* and *Rana*. Increasing relative proportions of heterospecifics reduced metamorph mass only in *Bufo*, indicating greater sensitivity to interspecific competition compared to *Rana*; the effect was reduced in the presence of fish. Our results show that undefended non-mimetic prey enjoy reduced predation with increasing relative abundance of chemically defended prey, which in turn suffer greater mortality with an increasing proportion of the undefended species. Associational resistance/susceptibility, driven by current assemblage composition, not by selection for resemblance, can shape the dynamics of mixed communities of defended and undefended prey in the absence of mimicry.

## Introduction

According to the basic definition of Batesian mimicry, the presence of a defended prey organism (model) increases the survival of undefended prey (mimics) that resemble the model due to a generalised aversive reaction in their shared predator (Ruxton et al. 2004). The evolution of mimicry assumes the adaptive resemblance of the mimic to the model in one or more sensory modalities of the predator (Dalziell and Welbergen 2016). In some systems, merely superficial similarity can provide substantial protection for the undefended species through imperfect mimicry (Edmunds 2000; Sherratt 2002; Lindström et al. 2004; Penney et al. 2012), which may result from relaxed selection in the mimics to resemble the models (Kikuchi and Pfennig 2013). When the probability of encountering a defended model prey is high, or its defences present very high costs to a potential predator, the latter’s optimal strategy is to avoid foraging on all prey that even moderately resemble the defended model (Sherratt 2002, Kikuchi and Pfennig 2010, 2013). Predator access to alternative food resources also increases survival chances for the imperfect mimics (Sherratt 2003, Lindström et al. 2004).

Even when the resemblance is poor, the concept of imperfect mimicry still assumes that one species evolves to resemble another, albeit to a limited extent (Sherratt 2002; Kikuchi and Pfennig 2013). However, prey lacking defences can also enjoy increased survival even when they are apparently not under selection to mimic the aposematic signal of the defended prey, or when the defended prey exhibit no clear aposematic signal to be imitated (Nelson et al. 2010; Nesbit et al. 2016). In experimental systems, undefended prey may benefit from the mere presence of defended prey in a shared habitat (Mappes et al. 1999, de Wert et al. 2012). Such indirect benefits may be explained by ‘associational effects’, a concept originally developed for and widely applied to relationships among plants sharing herbivores (Barbosa et al. 2009; Hambäck et al. 2014; Champagne et al. 2016) and seldom used to interpret animal interactions (but see Nesbit et al. 2016; Wooster et al. 2016). This concept assumes that consumers respond to collective properties of assemblages of resource organisms; consumption levels depend on resource species composition, resulting in the increased (associational resistance) or decreased (associational susceptibility) survival of the neighbouring species (reviewed in: Barbosa et al. 2009; Underwood et al. 2014). Associational effects encompass an array of indirect interactions between resource organisms mediated by their shared consumers, such as apparent competition (e.g. Holt and Kotler 1987; Holt and Lawton 1994), shared doom (e.g. Wahl and Hay 1995; Emerson et al. 2012), as well as some examples of indirect mutualism (e.g. Perry et al. 2004). Here we focus on the dynamics of resistance/susceptibility effects along a gradient of relative abundance of palatable to unpalatable prey in the apparent absence of mimicry. The ratio of defended to undefended prey is a key factor in systems with Batesian mimicry (Lindström et al. 1997, 2001), as it strongly affects the predator’s strategy (Sherratt 2011). We argue that in systems lacking mimicry, low or high ratios of chemically defended to undefended prey may result in ecologically significant mimicry-like associational effects (Mappes et al. 1999; de Wert et al. 2012; Nesbit et al. 2016). Importantly, both associational and mimicry relationships can be additionally complicated by competition between the co-occurring prey species (Barbosa et al. 2009; Pfennig and Kikuchi 2012).

In our experimental setup, we investigated a system where two animal species with similar morphology, but different in levels of chemical defences and the presence of aposematic signalling, shared a generalist predator with access to alternative prey. We aimed to investigate the occurrence of associational resistance/susceptibility along a gradient of relative proportions of tadpoles of two anurans, the common toad *Bufo bufo* and the common frog *Rana temporaria*, in the presence/absence of a fish predator. *B. bufo* tadpoles are unpalatable to fish due to chemical defences, while *R. temporaria* are highly palatable (Glandt 1983, 1984; Loman and Lardner 1995; Manteifel and Reshetnikov 2002; Kloskowski 2011). *B. bufo* tadpoles exhibit black colouration, considered conspicuous and aposematic (Peterson and Blaustein 1991; Griffiths and Foster 1998; Álvarez and Nicieza 2009), while the marbled colouration of *R. temporaria* tadpoles is assumed to be inconspicuous and cryptic (Nicieza 1999). Despite limited overlap in habitat requirements (Van Buskirk 2005), the two species consistently co-occur in a fraction of water bodies used for breeding (Babik and Rafiński 2001; Gazzola & Van Buskirk 2015), including those containing fish (Bardsley and Beebee 1998; Laurila 1998). As a predator, we used the common carp *Cyprinus carpio*, an omnivorous fish partly sympatric with *B. bufo* and *R. temporaria* which readily feeds on tadpoles and can attain a large body size, facilitating predation on a wide range of prey sizes (Kloskowski 2011).

We hypothesised that in assemblages with a fish predator, (1) a larger proportion of undefended *R. temporaria* would survive metamorphosis under conditions of an increasing initial proportion of chemically defended *B. bufo* (associational resistance), while (2) a lower proportion of *B. bufo* would survive metamorphosis under conditions of a decreasing initial proportion of *B. bufo* (associational susceptibility). Because these prey species are involved in asymmetric competition (Gazzola and Van Buskirk 2015; but see Laurila 2000b), our final hypothesis was that (3) the relative proportions of *B. bufo* and *R. temporaria* would differentially affect metamorphic traits of the surviving individuals in the presence/absence of fish, with *R. temporaria* imposing adverse effects on the competitively inferior *B. bufo* in the absence, but not the presence, of fish.

## Materials and methods

We collected amplexed *B. bufo* and *R. temporaria* from the breeding ponds in mosaic landscape of NW Poland (*B. bufo*: 52°32′58.80″N; 15°52′25.61″E; *R. temporaria*: 52°37′14.47″N; 15°50′36.84″E) and transferred them to the Experimental Station of Poznań University of Life Sciences (52°36′12.15″N; 15°50′17.35″E). Animals were placed in single-species pens containing semi-natural small ponds. The spawn and early stage larvae were reared in cages installed in one of the ponds. Adults that provided the spawn and metamorphs that survived the experiment were returned to their native habitat. Fish used in the experiment were carp fry (9–10 cm total length) raised in semi-natural ponds where they had no contact with anuran larvae. One week prior to the experiment, the fish were kept in interior fibreglass tanks, where they were fed ad libitum with commercial pelleted feed (35% total protein and 9% crude lipid).

The experimental setup consisted of 48 cages equally distributed among 12 square concrete basins (40 m^2^ each, 1.5 m max. depth). Each pond was independently supplied with river water (nutrient range values: 0.31–0.61 mg PO_4_^−^ L^−1^; 0.4–0.5 mg NO_3_^−^ L^−1^; total nitrogen 2.16–3.48 mg N L^−1^) that passed through fine-mesh screens installed at the water inlets. The cages were constructed from mosquito nets (fibreglass covered with PVC, mesh size 1 mm) stretched over a cubical (1 m^3^) steel frame and inserted 5–10 cm into the sandy pond bottom. The cages had open bottoms, but were covered on the top with a PVC net (mesh size 10 mm) to prevent colonisation by large insects. Each cage contained a refuge for the tadpoles (PVC mesh cylinder; 1000 mm × 100 mm; mesh size 10 mm). Water levels reached 75% of the cage heights.

To test the effects of varying proportions of the palatable and unpalatable species on their survival in the presence of fish, we used seven treatments, in which the proportions of *R. temporaria* to *B. bufo* ranged from 0 to 1, the total number of tadpoles remaining constant. Each pond hosted four replicates of the same treatment combined with fish presence (one carp per cage in fish treatments). Due to space limitations, single-species treatments were replicated only twice, i.e. one pond contained two single-species cages for each species, all without fish, and one pond contained an analogous setup but included fish. The experimental design is summarised in Table 1.

**Table 1:** Summary of the experimental design

Treatment (initial proportion of *B. bufo*)	N *B. bufo* tadpoles	N *R. temporaria* tadpoles	Fish	N replicates
0	0	40	Yes/no	2/2
0.05	2	38	Yes/no	4/4
0.25	10	30	Yes/no	4/4
0.5	20	20	Yes/no	4/4
0.75	30	10	Yes/no	4/4
0.95	38	2	Yes/no	4/4
1	40	0	Yes/no	2/2

Treatment is expressed as the proportion of chemically defended *B. bufo* tadpoles of the constant total number (*N* = 40) of tadpoles stocked per cage

The total initial number of tadpoles was 40 tadpoles m^−2^, which is within the natural densities of *R. temporaria* and *B. bufo* (Van Buskirk 2005; Gazzola and Van Buskirk 2015; Bókony et al. 2016). In the results section, treatments are expressed as the proportion of *B. bufo* of the total initial number of tadpoles in a cage (Table 1). When *B. bufo* reached Gosner stage 25 (Gosner 1960), tadpoles were randomly assigned to and stocked into the cages (05 May 2016). At this time, *R. temporaria* tadpoles were slightly larger than *B. bufo* tadpoles, reflecting natural size differences and priority effects (Bardsley and Beebee 1998). The carp were introduced into cages the following day. Commercial pelleted feed (ca. 10 g) were added to cages on a weekly basis as an alternative food for the fish. To balance the potential effect of extra food availability to tadpoles in treatments with fish, we added pellets to cages without fish as well. We assumed that the feed was available ad libitum because the bottoms of all cages retained a certain amount of uneaten feed throughout the entire experiment. Additionally, during the first week post-stocking, the cages were inoculated with zooplankton by adding 7 L of natural pond water. Metamorphs with completely resorbed tails (Gosner stage 46) were removed from the cages with a dip net and weighed to 0.01 g. The few tadpoles that did not complete metamorphosis by the end of the experiment (16 Jun 2016) were included in the survival analyses, but not in the body mass analyses.

We evaluated survival to metamorphosis, time to metamorphosis (number of days between stocking date and collection date), and metamorph mass among treatment combinations using generalised linear mixed models (GLMMs). We present full models, including interactions between fish presence and the initial proportion of toads in each treatment. However, models that were re-estimated after removing the non-significant interaction terms (with exclusion criterion *P* > 0.1) yielded the same conclusions. We analysed the survival within each cage using models with a probit link and binomial distribution. For each anuran species, the number of survivors to metamorphosis was treated as a binomial response, while the number of tadpoles stocked was the binomial denominator. Since the replicates per pond were not entirely independent, pond was entered as a random factor in the survival analysis. In the analyses of time to and mass at metamorphosis, cage nested within pond was entered as a random factor. We did not account for the effects of thinning because initial tadpole proportions and the number of metamorphs in cages were correlated. Similarly, in the models on metamorph mass, time to metamorphosis was not considered, as it was not independent from fish presence/absence. However, when entered separately into the models, it was not significant (*P* > 0.1). All analyses were performed in GenStat 15.0 (VSN, Hemel Hempstead, UK).

## Results

**Fig. 1:**
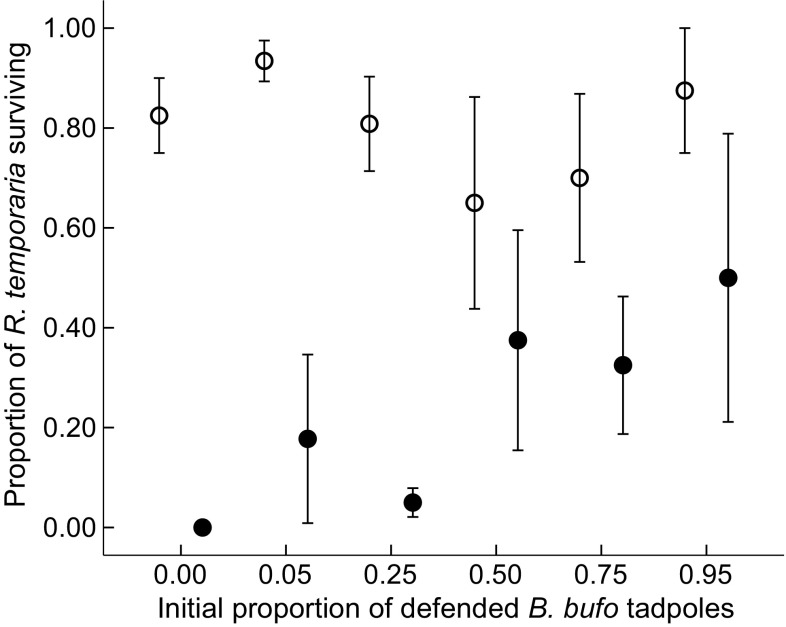
Proportion of undefended *Rana temporaria* tadpoles surviving to metamorphosis (mean ± SE) along the gradient of the initial proportions of chemically defended *Bufo bufo* in experimental tadpole communities. Empty circles indicate treatments in the absence of fish, filled circles indicate treatments with fish. Each treatment combination was replicated four times, except for the single-species treatment (initial *B. bufo* proportion = 0) replicated twice

**Fig. 2:**
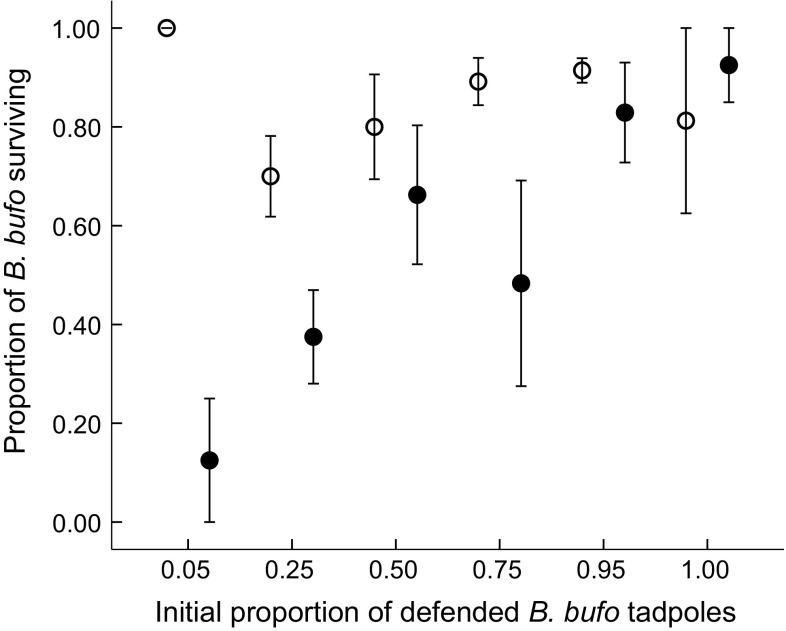
Proportion of chemically defended *Bufo bufo* tadpoles surviving metamorphosis (mean ± SE) along the gradient of their initial proportions in experimental tadpole communities. Empty circles indicate treatments in the absence of fish, filled circles indicate treatments with fish. Each treatment combination was replicated four times, except for the single-species treatment (initial *B. bufo* proportion = 1) replicated twice

**Fig. 3:**
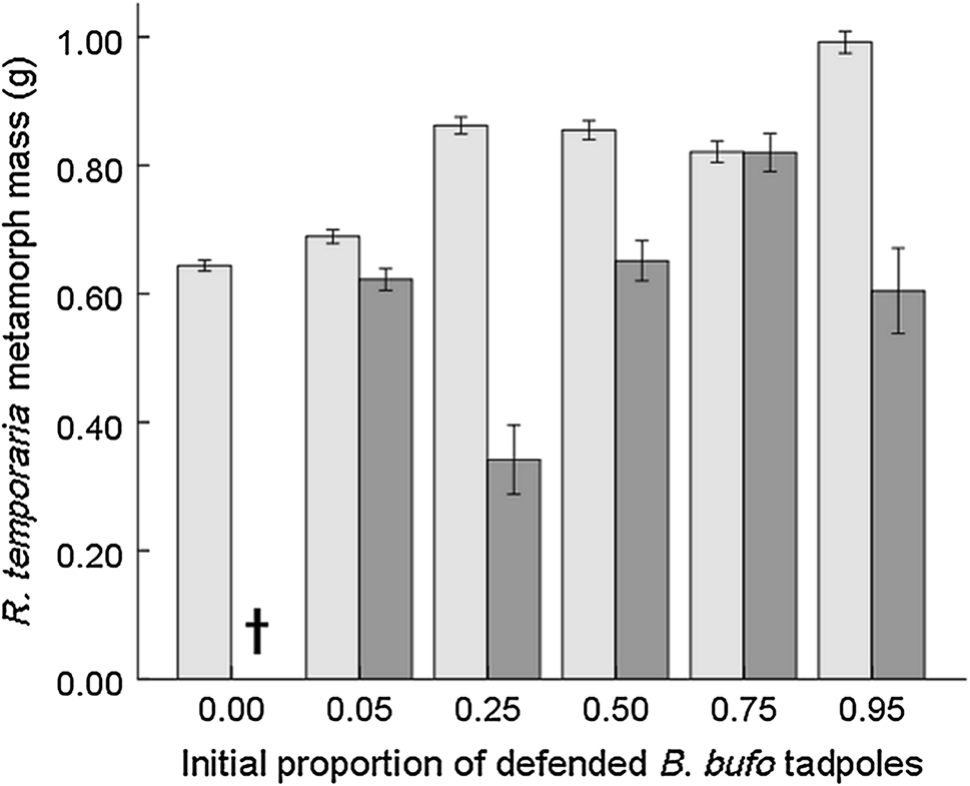
Metamorph mass (mean ± SE) of *Rana temporaria* (*N* = 470) along the gradient of the initial proportions of chemically defended *Bufo bufo* in experimental tadpole communities. Light-shaded bars indicate treatments without fish, dark-shaded bars indicate treatments with fish (for the number of replicates, see the caption of Fig. 1). In cages only stocked with *R. temporaria*, no tadpoles survived metamorphosis in the presence of fish (initial *B. bufo* proportion = 0; indicated with dagger)

**Fig. 4:**
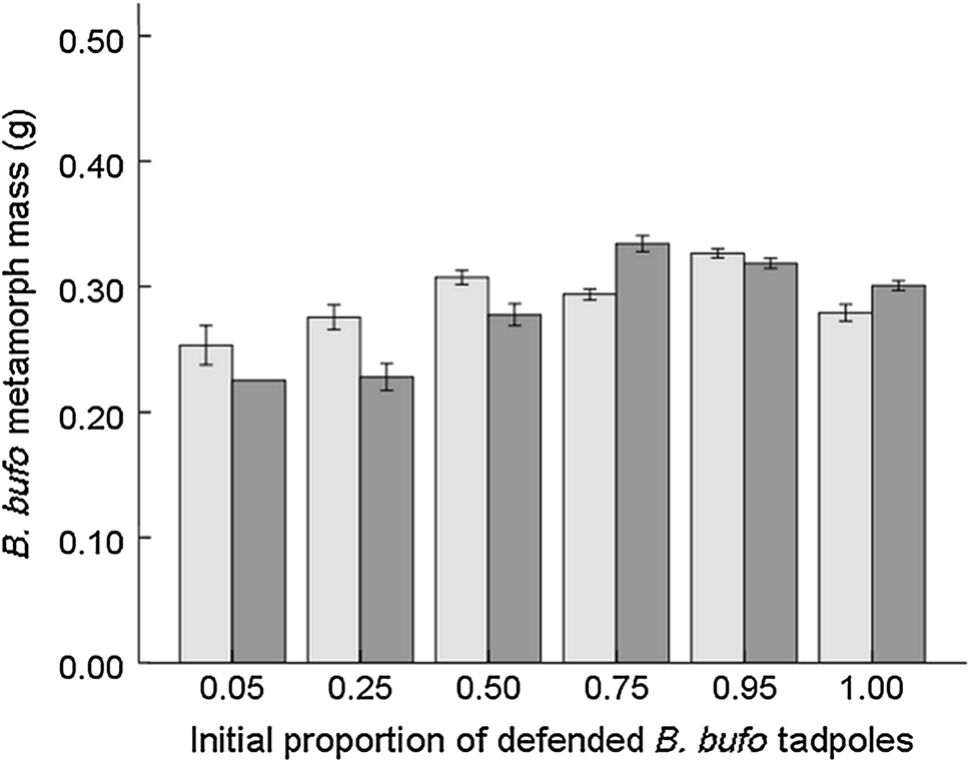
Metamorph mass (mean ± SE) of *Bufo bufo* (*N* = 719) along the gradient of their initial proportions in experimental tadpole communities. Light-shaded bars indicate treatments without fish, dark-shaded bars indicate treatments with fish (for the number of replicates, see the caption of Fig. 2)

Generally, fish reduced the survival of both *B. bufo* and *R. temporaria* tadpoles, although the effect was markedly stronger for *R. temporaria* (Table 2); in fish cages stocked exclusively with *R. temporaria*, all tadpoles were eliminated. The initial species proportion, as a main factor, did not affect *R. temporaria* survival (Fig. 1), while the survival of *B. bufo* tadpoles improved with their increasing initial proportion (Fig. 2). However, in the presence of fish, both *R. temporaria* (Fig. 1) and *B. bufo* (Fig. 2) tadpoles survived better with higher proportions of *B. bufo* per cage (Table 2). Fish prolonged time to metamorphosis in *B. bufo* (mean 34.6 ± 0.4 vs 35.7 ± 0.4 in fish cages; Table 3). *R. temporaria* mass at metamorphosis was lower in tadpoles exposed to fish, and increased with higher relative proportions of *B. bufo* (Fig. 3), but the treatment interaction was not significant (Table 4). In *B. bufo*, the main effect of fish presence was not significant, but mass at metamorphosis was positively affected by an increasing proportion of conspecifics (Fig. 4). The treatment interaction was only marginally significant (Table 4).

**Table 2:** GLMMs (binomial error structure, probit link) of the survival of *B. bufo* and *R. temporaria* tadpoles to metamorphosis

Species	Fixed factor	*F*	*df*	*P*	Effect (± SE)
*R. temporaria*	Fish	36.20	7.6	< 0.001	− 2.056 (± 0.315)
	Initial proportion of *B. bufo*	1.86	9.6	0.203	− 0.592 (± 0.655)
	Initial proportion of *B. bufo ×* fish	7.21	9.6	0.024	2.514 (± 0.936)
*B. bufo*	Fish	7.59	7.3	0.027	− 0.528 (± 0.248)
	Initial proportion of *B. bufo*	9.04	10.1	0.013	0.264 (± 0.545)
	Initial proportion of *B. bufo* × fish	5.26	10.1	0.044	1.772 (± 0.772)

Fish presence/absence, initial proportion of *B. bufo* (0; 0.05; 0.25; 0.5; 0.75; 0.95; 1) and their interaction were fixed factors. Pond was included as a random factor. Standard errors of coefficients are shown in parentheses; for the “fish” factor, standard errors of differences are reported

**Table 3:** GLMMs of time to metamorphosis (days) of *B. bufo* and *R. temporaria* tadpoles using fish presence/absence, initial proportion of *B. bufo* (0; 0.05; 0.25; 0.5; 0.75; 0.95; 1) and their interaction as explanatory variables

Species	Fixed factor	*F*	*df*	*P*	Effect (± SE)
*R. temporaria*	Fish	2.94	30.8	0.097	1.364 (0.354)
	Initial proportion of *B. bufo*	3.31	31.7	0.078	2.127 (0.874)
	Initial proportion of *B. bufo* × fish	2.88	36.0	0.098	− 2.877 (1.695)
*B. bufo*	Fish	5.58	38.9	0.023	1.138 (0.510)
	Initial proportion of *B. bufo*	1.18	43.3	0.283	− 0.825 (0.889)
	Initial proportion of *B. bufo* × fish	0.01	43.6	0.904	0.174 (1.435)

Cage nested within pond was included as a random factor. Standard errors of coefficients are shown in parentheses; for the “fish” factor, standard errors of differences are reported

**Table 4:** GLMMs of *B. bufo* and *R. temporaria* mass (g) at metamorphosis with fish presence/absence, initial proportion of *B. bufo* (0; 0.05; 0.25; 0.5; 0.75; 0.95; 1) and their interaction as explanatory variables

Species	Fixed factor	*F*	*df*	*P*	Effect (± SE)
*R. temporaria*	Fish	16.67	30.0	< 0.001	− 0.248 (0.060)
	Initial proportion of *B. bufo*	18.10	30.5	< 0.001	0.255 (0.080)
	Initial proportion of *B. bufo* × fish	0.29	33.0	0.591	0.093 (0.150)
*B. bufo*	Fish	0.48	34.0	0.492	− 0.003 (0.012)
	Initial proportion of *B. bufo*	19.20	42.6	< 0.001	0.048 (0.022)
	Initial proportion of *B. bufo* × fish	3.92	42.7	0.054	0.069 (0.035)

Cage nested within pond was included as a random factor. Standard errors of coefficients are shown in parentheses; for the “fish” factor, standard errors of differences are reported

## Discussion

We provided experimental evidence that, during the larval stage, both undefended and defended prey survived fish predation better with higher proportions of defended prey in the assemblage. Our results show that *R. temporaria* tadpoles enjoyed increased resistance to predation with increasing abundance of chemically defended heterospecifics, while *B. bufo* suffered increased susceptibility to predation with increasing abundance of undefended frog tadpoles. Admittedly, the effect of the relative proportions of the two species is difficult to separate from the effect of the absolute density of the chemically defended *B. bufo* on its self-protection (cf. Rowland et al. 2010). Additionally, the negative effect on *B. bufo* of the relative proportion of *R. temporaria* regardless of fish presence shows that interspecific competition may contribute to *B. bufo* mortality as well.

The potential mechanism explaining our results requires a change in the predator’s behaviour induced by the composition of the prey assemblage, i.e. a prey-induced trait-mediated indirect interaction (Werner and Peacor 2003; Nomura et al. 2011). The proportion of the chemically defended tadpoles in the assemblage may equate to the level of predator experience with such prey. This leads to generalised aversion to all tadpoles when defended tadpoles are often encountered (Nelson et al. 2010; Caller and Brown 2013) and killing of defended tadpoles when they are rarely encountered (Kruse and Stone 1984; Nomura et al. 2011). This generalised aversion may be prevented if the fish use a taste-and-refuse strategy to differentiate between prey types (Nelson et al. 2011). However, if this strategy is not profitable, the effect should occur and persist, e.g. when chemically defended prey are abundant and alternative (here: non-tadpole) food sources are available (Nonacs 1985; Holen 2013). As a consequence, any significant survival rate of undefended tadpoles requires the presence of relatively numerous defended tadpoles, unless the undefended species occurs at densities exceeding the predator’s capacity for consumption (cf. Sherratt 2003).

Our results suggest that when unequally defended prey types share basic morphological features but otherwise bear limited resemblance, they can indirectly affect each other’s survival via a shared predator. Considering the patterns of prey survival, the effect is analogous to imperfect Batesian mimicry (Lindström et al. 2004). Very slight similarities between prey species may affect predator behaviour and lead to emergence of mimicry-like effects (Holling 1965). For a predator, investment into discrimination abilities between unevenly defended prey from a single ‘class’ (here, tadpoles of different species) can be unprofitable, e.g. when the undefended prey are rare or consumption of the defended prey is costly (Sherratt 2002; Kikuchi and Sherratt 2015). However, although the investigated system behaves in line with the predictions of the models of imperfect mimicry, we argue that it does not fit into its definition. There is no indication that the undefended *R. temporaria* mimics the aposematic signal (black body colouration) of the defended *B. bufo*. Instead, the two species share a non-signalling cue (body shape). Although cue-based mimicry is widespread in natural systems (Jamie 2017), the similar appearance of the two species used in our study is rather a consequence of ecological and phylogenetic constraints, as the ‘generalised tadpole morphology’ is common among anurans from temperate latitudes (Altig and McDiarmid 1999). Consequently, we prefer to interpret the outcomes of the study within the more general framework of associational effects (Underwood et al. 2014). In contrast to mimicry, which requires several conditions to be met (Ruxton et al. 2004; Ruxton and Schaefer 2011; Dalziell and Welbergen 2016), the concept is based merely on the assumption that consumers responding to collective properties of assemblages of resource organisms, i.e. consumer effects, on different spatial scales strongly depend on resource community composition (e.g. Kuijper and Bakker 2008; Plath et al. 2012; Underwood et al. 2014). Although here we focus on the effects resulting from unequal levels of defence against predators, the concept may be useful for tracking various interactions that are rather a by-product of current community composition than a consequence of selection for mimicking signals of another species. For animal communities, examples include ‘accidental mimicry’ between originally allopatric species or interactions between defended and undefended prey sharing an undiscriminating predator (Nelson et al. 2010; Nesbit et al. 2016; Wooster et al. 2016; see also Robertson 2013, for ‘social traps’ resulting from coincidental visual resemblance in reef fish). Importantly, associational effects related to unequal defences in co-occurring species may in fact function as an incipient stage of the evolution of actual mimicry complexes (Mappes et al. 1999; de Wert et al. 2012).

In our study, metamorph size of *R. temporaria* increased with a decreasing proportion of conspecifics in the system, while metamorph size of *B. bufo* decreased with an increasing proportion of heterospecifics, consistently with the hypothesis of asymmetric competition between tadpoles of the two species (Gazzola and Van Buskirk 2015). Such competitive asymmetry is likely to be driven by tadpole size differences (Richter-Boix et al. 2004), as *R. temporaria* usually breeds earlier in the season than *B. bufo* (Tryjanowski et al. 2003) and its tadpoles remain larger during the development (Bardsley and Beebee 1998; Gazzola and Van Buskirk 2015). However, the effects were reduced in the presence of carp, indicating that fish mitigated interspecific competition between tadpoles (see also Bardsley and Beebee 1998) through thinning and, presumably, behavioural changes induced in the competitively superior *R. temporaria* tadpoles. In cages with fish, metamorph mass of *R. temporaria* was lower, presumably because its tadpoles reduce their activity in the presence of predators (Laurila et al. 1997; Laurila 2000a; Lardner 2000; Maag et al. 2012), leading to reduced food intake (Lardner 2000; Relyea 2007). In *B. bufo*, a species whose tadpoles reduce their activity only slightly in the presence of predators (Laurila et al. 1997; Maag et al. 2012), metamorph mass was unaffected by fish. Predators can also induce changes in the timing of metamorphosis in anurans, but the magnitude and direction of the effect are variable (Relyea 2007; Wells 2007). *B. bufo* needed more time to reach metamorphosis when fish were present, but the prolongation of the larval period averaged only about 1 day.

Associational effects are likely to be widespread in larval anuran communities where chemically defended species co-occur with undefended species. In natural environments, some *R. temporaria* may spawn in ponds inhabited by fish (Laurila and Aho 1997; Laurila 1998; Van Buskirk 2005), even though fish eliminate most of *R. temporaria* tadpoles (Bardsley and Beebee 1998; Laurila 1998). Through associational protection, *B. bufo*’s presence may increase the survival of *R. temporaria* tadpoles in sites that otherwise would act as population sinks. As fish introductions pose a risk to amphibian populations worldwide (Kats and Ferrer 2003), the potential associational resistance enjoyed by species vulnerable to fish in the presence of chemically defended heterospecifics could provide community-level conservation benefits (Hartman et al. 2014). Contrastingly, the presence of palatable tadpoles may increase vulnerability to predation in defended anuran larvae. Thus, *B. bufo*’s tendency to avoid heterospecific tadpoles and form single-species aggregations (Griffiths and Denton 1992; Bardsley and Beebee 1998) could be viewed as an adaptation counteracting associational susceptibility. Elevated toxin levels may act similarly, but recent findings show that *B. bufo* toxicity increases in the presence of both conspecific and heterospecific tadpoles (Bókony et al. 2016, 2017).

In conclusion, our results show significant reciprocal indirect effects on survival between chemically defended and undefended amphibian larvae mediated by a shared predator. We demonstrated that, given certain proportions of the two tadpole species, under conditions of availability of alternative food, the effects can persist until metamorphosis. Given that these effects depend on assemblage composition and are not driven by selection for resemblance, associational resistance/susceptibility can affect the dynamics of assemblages of defended and undefended species where mimicry is not present. We encourage future studies on unequally defended prey sharing a predator to use proportional gradients of prey, since the relative composition of resource species is central to the character of interactions in a wide array of predator–prey systems (Holt and Lawton 1994; Underwood et al. 2014).
